# Reading as a Predictor of Complex Syntax. The Case of Relative Clauses

**DOI:** 10.3389/fpsyg.2019.01450

**Published:** 2019-07-10

**Authors:** Luca Cilibrasi, Flavia Adani, Ianthi Tsimpli

**Affiliations:** ^1^Department of Theoretical and Applied Linguistics, University of Cambridge, Cambridge, United Kingdom; ^2^Faculty of Arts, Charles University, Prague, Czechia; ^3^Department of Education and Psychology, Free University of Berlin, Berlin, Germany

**Keywords:** relative clauses, reading, acquisition, predictors, complex syntax

## Abstract

**Background:**

The current study aims at better characterizing the role of reading skills as a predictor of comprehension of relative clauses. Well-established cross-linguistic evidence shows that children are more accurate in the comprehension of subject-extracted relative clauses in comparison to the object-extracted counterpart. Children with reading difficulties are known to perform less accurately on object relatives at the group level compared to typically developing children. Given that children’s performance on reading tasks is shown to shape as a continuum, in the current study we attempted to use reading skills as a continuous variable to predict performance on relative clauses.

**Methods:**

We examined the comprehension of relative clauses in a group of 30 English children (7–11 years) with varying levels of reading skills. Reading skills varied on a large spectrum, from poor readers to very skilled readers, as assessed by the YARC standardized test. The experimental task consisted of a picture-matching task. Children were presented with subject and object relative clauses and they were asked to choose one picture - out of four - that would best represent the sentence they heard. At the same time, we manipulated whether the subject and object nouns were either matching (both singular or both plural) or mismatching (one singular, the other plural) in number.

**Results:**

Our analysis of accuracy shows that subject relatives were comprehended more accurately overall than object relatives, that responses to sentences with noun phrases mismatching in number were more accurate overall than the ones with matching noun phrases and that performance improved as a function of reading skills. Within the match subset, while the difference in accuracy between subject and object relatives is large in poor readers, the difference is reduced with better reading skills, almost disappearing in very skilled readers.

**Discussion:**

Beside replicating the well-established findings on the subject-object asymmetry, number facilitation in the comprehension of relative clauses, and a better overall performance by skilled readers, these results indicate that strong reading skills may determine a reduction of the processing difficulty associated with the hardest object relative clause condition (i.e., match), causing a reduction of the subject-object asymmetry.

## Introduction

Children with reading difficulties often show problems that extend beyond reading itself (Savage and Frederickson, 2006) and surface in other domains of language, such as syntax or verbal working memory (Robertson and Joanisse, 2010). Since the reading skills of pupils shape as a continuum (Pennington, 1995; Snowling, 2013), we aim to establish in this study whether a continuous measure of reading can be used to predict performance in a widely investigated syntactic domain: relative clauses. The choice of using a continuous measure of reading (rather than separating children into groups) has two advantages: First, it is a more faithful representation of the population (due to the aforementioned continuous distribution of reading skills). Second, it offers the possibility of using a model where reading skills can be used as a predictor.

The paper consists of the following steps: in the introduction, we present previous literature that uses reading as a predictor of complex syntax and we provide an introduction to relative clauses and to their syntactic analysis. We then proceed with the presentation of participants, methods, and procedure. We then present the results, followed by discussion and conclusion.

### Reading as a Predictor of Complex Syntax

The impact of reading skills on the acquisition of complex syntactic structures was investigated in several studies. In seminal work, Nation and Snowling (2000) showed that poor readers are less accurate in the completion of tasks that require high syntactic awareness. In their experiment, primary school children of different grades were asked to reorder sentences in which the words were scrambled. Poor readers were revealed to be consistently less accurate than typically developing (TD) children.

In some cases, the relation between poor reading and poor morphosyntactic skills may be the consequence of comorbidity between a reading and a syntactic impairment. Nation et al. (2004) showed that poor readers are more likely to meet the diagnostic criteria for specific language impairment (SLI) than children with no reading difficulties, and tend to be less accurate in standardized morphosyntactic assessments. A similar finding is reported by McArthur et al. (2000), who attests the comorbidity of dyslexia and SLI to have a prevalence of 50%. However, the relation between reading and language skills holds also within the TD spectrum: in a large sample study of 180 TD children in grades 3–5, Goff et al. (2005) showed that children with strong vocabulary skills are more likely to be good readers (only vocabulary skills were used as a prompt of language). Along similar lines, Cain (2007) investigated the relation between reading skills and syntactic awareness (with a word scrambling task): while vocabulary was a strong predictor of reading comprehension, syntactic awareness revealed to be a strong predictor of word reading (see also Mokhtari and Thompson, 2006). Interestingly, in younger children (4-year-olds) that are still not able to read, morphosyntactic performance is predicted by shared reading with their parents (Sénéchal et al., 2008).

Reading and working memory are often associated, and several studies have tried to better characterize the relation between the two. Leather and Henry (1994), for example, showed that working memory plays a crucial independent role as predictor of reading accuracy.

In a longitudinal study, Cain et al. (2004) showed that 8-, 9-, and 11-year-olds’ reading skills are predicted independently by working memory, reading comprehension, and inferencing. This result is consistent with data showing that children with reading difficulties experience problems with non-word repetition (a measure of verbal working memory), as it is shown in Gathercole et al. (2006).

In a study conducted on 9-year olds previously tested in pre-school, Muter and Snowling (1998) better defined the differences between predictors and precursors. At age 9, the predictors of reading accuracy were vocabulary, reading rate, and phoneme awareness. Instead, the variables that measured in pre-school better predicted reading accuracy at 9 (i.e., the precursors) were phoneme deletion, letter knowledge and, crucially, working memory (non-word repetition). This finding is consistent with Goff et al. (2005), who showed that, when measured concurrently, language measures can predict reading accuracy more reliably than working memory can.

Some studies focussed specifically on the comprehension of relative clauses in children with reading difficulties. Seminal work by Bar-Shalom et al. (1993) shows that problems with object relative clauses are observed in children with dyslexia, and are more evident than those observed in typically developing children. The result was replicated cross-linguistically in French (Casalis et al., 2013), Hebrew (Leikin and Bouskila, 2004), and Italian (Arosio et al., 2017). In a study that directly addressed the relation between working memory and comprehension of relative clauses in children with dyslexia, Robertson and Joanisse (2010) showed that children with dyslexia perform worse overall than controls in the comprehension of object relatives. In this study, the author manipulated the length of sentences and the time occurring between presentation of the sentence and presentation of the pictures, in order to manipulate working memory loads. Their results crucially show that the difficulties of children with dyslexia are restricted to the trials with a high working memory load.

### Relative Clauses

The acquisition of syntax follows several developmental steps, and it is clear that not all structures are acquired at the same time during development, and not all structures are comprehended and produced with the same accuracy (Tsimpli, 2014; Guasti, 2017). This paper focuses on a structure that is acquired late (Tsimpli, 2014): relative clauses. The comprehension of relative clauses is a widely studied phenomenon and the asymmetry in the comprehension of subject relatives (e.g., show me the lion that washes the elephant) vs. object relatives (show me the lion that the elephant washes) is one of the most consistent results in the psycholinguistic literature. The result was reported in different languages, such as English (Brown, 1972; Keenan and Comrie, 1979), Italian (Contemori and Belletti, 2014), Hebrew (Friedmann et al., 2009), Chinese (Hu et al., 2016), Cypriot Greek (Theodorou and Grohmann, 2012), Spanish (Betancort et al., 2009), and it was reported in TD children (ibid), children with SLI (Adani et al., 2014), children with Dyslexia (Cantiani et al., 2013; Casalis et al., 2013; Arosio et al., 2017).

A formal explanation for this pattern of results is offered by the concept of Relativised Minimality (Rizzi, 1990) and its more recent development, featural Relativised Minimality (Garraffa and Grillo, 2008; Friedmann et al., 2009; Grillo, 2009; Rizzi, 2013). According to this proposal, the difficulties with structures such as object wh-questions and object relative clauses are due to the disruption of so-called *local relationships*. Local relationships are what occur when two elements are (1) co-referential and (2) one c-commands the other.

(1) In non-canonical word orders, some empty positions are filled by traces of the referent noun, creating co-referentiality between a noun and its trace. For example, in the sentence: “the house he bought is nice,” the verb “to buy” is missing an overt object. It is assumed that the empty position is filled by a psychological trace that is co-referential with the NP “the house,” leading to: The house he bought <t> is nice.

(2) The notion of c-command, instead, refers to the hierarchical position of elements in a sentence. Specifically, an element A c-commands an element B when it is in a higher position in the sentence, and the first node that dominates A also dominates B.

A local relationship can be *disrupted* when a third element structurally intervenes. A specific configuration is required for intervention to apply: given A and B in a local relation, Z intervenes if it c-commands B and is c-commanded by A. The more structurally and featurally similar Z is to A and B, the stronger the effects of intervention.

In object relative clauses, the NP of the main clause is in a local relationship with its trace, as in:

(3) The dog that the fish is splashing <t> is sitting on the ground

In fact, the dog is co-referential with its trace and it c-commands it. Now, in this type of configuration, the NP <the fish> does meet the requirements for intervention: it is of the same structural type (it is another NP), it c-commands <t> and it is c-commanded by <the dog>.

Object relatives, such as (3), then, undergo intervention. Subject relatives, instead, do not undergo intervention, as it can be observed in (4).

(4) The dog that < t > is splashing the fish is sitting on the ground

Also in (4) there is a local relationship between the dog and <t>. In this case, however, the other NP <the fish> cannot intervene: Despite being of the same structural type, it does not appear in the required syntactic configuration, since it does not c-command <t>.

According to Garraffa and Grillo (2008) and Grillo (2009), sentences that undergo intervention present problems for people with language disorders because in these populations the access to features is limited, and as such sentences that are grammatical but non-canonical generate strong intervention effects.

Difficulties with sentences that undergo intervention are reported in various forms in different populations. In typically developing children, structures with intervention are acquired later than structures without intervention (Guasti, 2017). In children with language impairment, structures with intervention remain problematic even when non-intervention structures are acquired (Adani et al., 2014). In adults with aphasia, structures with intervention are affected while the non-intervention structures are spared (Garraffa and Grillo, 2008). According to Garraffa and Grillo (2008), processing limitations are at the basis for these findings: in subjects with processing constraints, the access to the features needed to parse intervention sentences may be interrupted or limited, and as such these populations may struggle.

A possible explanation for the asymmetry between subject and object relatives is thus that intervention effects arise from processing limitations, and the consequent limits to the access to features (Garraffa and Grillo, 2008). Working memory may be at the core of these limitations. According to Tsimpli (2014), the comprehension of relative clauses relies on language external and language internal resources, since large working memory resources are necessary to parse structures with long dependencies and interference. Object relatives may not be difficult because the structure is challenging *per se*, but rather because parsing such a complex structure requires a heavy involvement of working memory. In summary, the asymmetry between subject and object relatives may be explained within this account: structures with a high degree of intervention may pose a challenge for working memory, since access to features is crucial in keeping the filler-gap dependency when there is an interfering element.

According to an alternative proposal (MacDonald, 2013), the difficulties speakers encounter with the comprehension of object relatives is to be found in the fact that these are less frequent in the input than subject relatives. According to McDonald, these structures are produced less frequently because of structural complexity, and, as a consequence of scarcity in the input, perception is affected too. In other words, while the difficulties in production arise from processing constraints, the difficulties in perception are a consequence of input (Riches and Garraffa, 2017). When looking at both written and spoken corpora, the frequency of object relatives is indeed considerably smaller overall than that of subject relatives (Roland et al., 2007). However, despite this general pattern, the frequency of relative clauses interacts in non-trivial ways with modality, as investigated in a large corpus study by Roland et al. (2007): first, when considering both subject and object structures, relative clauses appear more frequently in written than in spoken language. However, when looking at the proportion of subject vs. object relatives in a given modality, object relatives appear to be more frequent in spoken language than in written language. As Roland et al. (2007) note, this effect is driven by one specific type of object relative, the one with the structure: [Inanimate-NP + (that) + pronoun + V]. This type of structure crucially differs from the structure used in our task, where two full animate NPs are used. As such, a more relevant fact to consider in our study is that non-canonical word orders tend to be more frequent in written than in spoken language (Purcell-Gates, 2001). Where data are available, relative clauses with full NPs are simply shown to be very rare (Kidd et al., 2007), and object relatives with full NPs are considerably rarer than subject relatives (Heider et al., 2014; Adani et al., 2017).

## Hypothesis and Predictions

Our hypothesis is that reading skills will be related to the comprehension of relative clauses, and that children with poor reading skills will experience more difficulties with complex syntax. We predict that performance on relative clauses will be modulated by reading, so the effect of complexity will become gradually larger in correspondence of gradually lower reading skills. Though with a different grouping and analysis, our predictions are based on previous results investigating reading and relative clauses, such as Bar-Shalom et al. (1993), Leikin and Bouskila (2004), Casalis et al. (2013), and Arosio et al. (2017). Our predictions are consistent with two observations:

(A) Children with better reading skills tend to have more experience with non-canonical word orders (MacDonald, 2013).
(B) The enhanced working memory skills of good readers allow for better access to features (and access to features helps the processing of intervention structures [Garraffa and Grillo, 2008)].

The fact that these two observations come from different theoretical approaches (input vs. structural) should not imply that they contradict each other, as frequency and structural effects can point to the same prediction (Adani et al., 2017).

## Participants

Thirty children were recruited in the Cambridge (United Kingdom) area, through primary schools that agreed to participate in the project and through colleagues at the university. The mean age of the children was 9;04, SD 1;02, range 7;05 to 11;07, 14 female and 16 male. All children were monolingual and none of the children was diagnosed with language or developmental impairments. Children were assessed with a non-word repetition task, the Children’s Test of Non-word Repetition (Gathercole et al., 1994), and with a test of non-verbal intelligence, the Colored Progressive Matrices (Raven, 1998). All children performed within the norms for their age in these tests. All tests used were coded using percentiles, so that age variation would not affect the result^1^. Furthermore, children were assessed with a standardized reading test, the York assessment of reading and comprehension (Snowling et al., 2009). The YARC is a reading assessment in which children are asked to read short passages while the researcher measures their reading time and their decoding mistakes (on a separate answer sheet). After completing the passage, children are asked 8 comprehension questions. The YARC offers 3 measures of reading for each child: decoding, fluency (rate) and comprehension. In our study we decided to use reading rate as a measure of reading skills. This choice is motivated by two factors: first, reading rate was shown to be significantly more effective than decoding as a clinical marker of dyslexia (Serrano and Defior, 2008). Second, comprehension questions in the YARC partly tap into pragmatic skills and lexical knowledge, and, since this was not the object of our study, we did not want these effects to affect our result. Since reading variability was a crucial aspect in the design of this study, we asked teachers to select from their class pupils that would show a wide spectrum of reading skills.

Before collecting the data, the proposal for the study was submitted to the University of Cambridge Ethics Committee and it was given favorable opinion to proceed. Parents of the children signed a consent form that is now stored in a secure location. Raw data is anonymized and stored in a secure location.

## Materials and Methods

The task used in this study was developed by Adani et al. (2014). In this task there are two main manipulations: type (subject relatives vs. object relatives) and match (noun phrases either matching or mismatching in number). The manipulation of type reflects classic work on relative clauses (Brown, 1972; Friedmann et al., 2009). In addition to the manipulation of type, the sentences were manipulated so that the noun phrases would either match or mismatch in number. The two nouns involved in the action could be both singular or both plural (match), or one singular and one plural (mismatch).

There were thus 4 cross-conditions:

SR, Mismatch:
The cats that are combing the rabbit have entered the box.
OR, Mismatch:
The cats that the rabbit is combing have entered the box.
SR, Match:
The cat that is combing the rabbit has entered the box.
OR, Match:
The cat that the rabbit is combing has entered the box.

**FIGURE 1:**
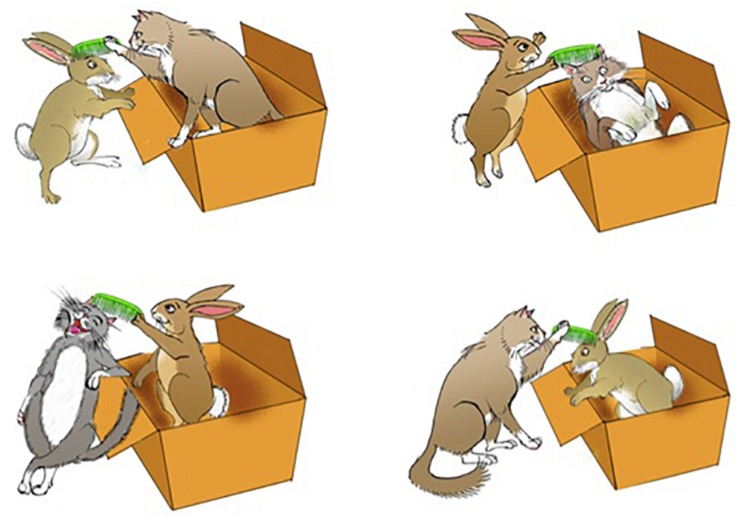
Example pictures for one trial of the experimental task. Permission to use this picture was granted by Prof. Maria Teresa Guasti, University of Milan Bicocca.

The task consist of 12 trials for each condition, totalling 48 sentences. There were 4 practice trials and no fillers. Subjects were presented aurally with a sentence of the type above and were asked to choose one of the four pictures in Figure 1. Sentences were previously recorded by a native female English speaker. Pictures relate to the sentences in that (1) the theta-roles of the embedded clause are either appropriate or inverted, and (2) the subject of the embedding clause is either well-represented or misrepresented. Hence, only one picture is an appropriate answer to each sentence: the one in which the theta-roles of the embedded clause are appropriate, and the subject of the embedding clause is well-represented. For example, given “The rabbit that is combing the cat has entered the box,” only the picture in the bottom-left represents the embedded verb (to comb) with the appropriate theta-roles (the rabbit combs the cat) AND the subject of the embedding clause appropriately (with a rabbit inside the box). See Figure 1.

## Procedure

Schools allowed for 1-h testing slots for each child. In each given school day, we thus tested a maximum of four children, and testing took place over several weeks. Some of the parents preferred to do the testing in the afternoon at the university, and testing was thus completed at the Psycholinguistics Lab of the Department of Theoretical and Applied Linguistics, University of Cambridge. The order of testing was as follows: first, children were assessed with the YARC, followed by the CNRep and the Raven’s matrices. Then, they were assessed with the syntactic task (Adani et al., 2014) and with a morphological task (Cilibrasi et al., 2019) that we do not present in this paper.

The syntactic task was presented using Microsoft PowerPoint. First, a few practice slides were presented to ensure that the child understood the task. Then, the child was presented with the experimental items that were randomized using an extension of PowerPoint (Tushar Mehta Randomizer). Each figure on the screen was identified by a number (from 1 to 4). The child named which number they deemed appropriate and the researcher completed a form accordingly. An index at the top of each slide guided the researcher in the completion of the form.

## Results

**FIGURE 2:**
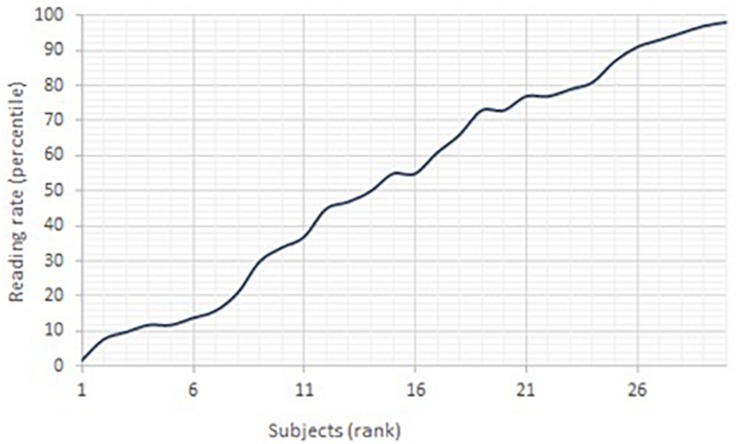
Line-graph of reading rate – rank.

The pre-selection of the children operated by the teachers was a successful strategy, since there is a strong linear correlation between rank and reading percentile, and the scores span from very low (1 child meets the diagnostic criteria for Dyslexia, and 6 would fall in the category of poor readers) to very high (indicating exceptionally skilled readers). The distribution can be observed in Figure 2 below:

Considering that reading performance of pupils distribute on a continuum (Crisp and Lambon Ralph, 2006; Snowling, 2013), we tried to capture the effects of reading on syntax by using continuous rather than categorical measures of reading proficiency. In other words, instead of dividing the sample into children with dyslexia, poor readers and TDs, we kept all the children in one sample and used reading as a predictor in the model. First, descriptive statistics were run on the experimental task (Table 1) and on the background task (Table 2).

**TABLE 1:** Descriptive statistics syntactic task.

**Match**	**Type**	**Mean (proportion of correct)**	**SE**
M	OR	0.65	0.2
MM	OR	0.7	0.2
M	SR	0.73	0.2
MM	SR	0.85	0.2

*M, match; MM, mismatch; OR, object relative; SR, subject relative; SE, standard error.*

**TABLE 2:** Descriptive statistics reading and memory tasks (percentiles).

	**Reading accuracy**	**Reading rate/speed**	**Reading comprehension**	**Working memory (CNRep)**
Mean	55.43	53.2	68.2	48.83
SD	29.92	31.39	23.25	26.02

Then, based on previous studies, an analysis of collinearity between working memory and reading was conducted in order to decide whether to include working memory in the model. We found a significant correlation between reading rate and working memory, *r* = 0.77, *p* < 0.001, and we decided thus to run the analysis without working memory, following the advice of Schroeder et al. (1990) to exclude one of the variables when dealing when collinearity. We analyzed the proportion of correct responses with generalized linear mixed models within the lme4 package (Bates et al., 2015). A logistic function (lgmer) was used because the dependent variable (accuracy) could assume two values: correct and incorrect. To ensure that working memory was not better than reading as a predictor we compared a full model in which working memory was included but reading was not to a full model in which reading was included but working memory was not, using a default random structure of (1|part) + (1|item). The result shows that the model with reading and no working memory is a better fit [as shown by smaller values of BIC and AIC after running the anova() command].

We then followed a well-established procedure to find the best random structure (Baayen et al., 2008; Pérez et al., 2016): A full model with all factors and interactions was used as starting point: correct ∼ reading^*^type^*^match. Keeping this full fixed structure, several random structures were compared using the anova() command. The full list of compared random structures is available in the Appendix. The most explanatory random structure was: (type|part) + (1|item), as shown by the smallest AIC value.

The final model was thus:

MFinal < - correct ∼ (yarcRATE.cent^*^type^*^match + (type|part) + (1|item), data = ukstudy2, family = binomial, control = glmerControl(optimiser = “bobyqa,” nAGQ(10), na.action(=na.omit).

Since by default r assigns one value of each categorical variable to the intercept, and since this makes the reading of the interactions complex, we releveled the categorical variables (type and match) using the function sliding contrast (from the MASS library). Levels were assigned with the following values: +, subject relatives; −, object relatives; +, mismatch; −, match.

The LME revealed a 3-way interaction between match, type and reading, as well as main effects of type and reading. The signs of the estimates can be used to understand the direction of the main effects, with no need for *post hoc* (Jaeger, 2008; Field, 2013). Subject relatives were comprehended more accurately overall than object relatives, better performance in reading corresponded to a better performance with relative clauses, and sentences with mismatch in number were comprehended more accurately overall than sentences with match in number (Table 3).

**TABLE 3:** Full model output.

**Fixed effects**	**Estimate**	**SE**	***Z*-value**	***p*-value**
Intercept	1.068	0.207	5.151	< 0.001
Match 2-1	0.494	0.211	2.33	0.019
Type 2-1	0.536	0.242	2.213	0.026
Reading	0.014	0.006	2.43	0.015
Match 2-1: Type 2-1	0.47	0.423	1.112	0.266
Match 2-1: Reading	0.003	0.004	0.83	0.406
Type 2-1: Reading	–0.002	0.005	–0.409	0.682
Match 2-1: Type 2-1: Reading	0.02	0.008	2.312	0.021

*Gray shade indicates significant result.*

Following the three-way interaction, we decided to split the datafile in match vs. mismatch sentences. The choice of using match (rather than type) as a dividing variable is motivated by our interest in observing the asymmetry between subject and object relatives, and using type as a dividing variable would have avoided a direct comparison between subject relatives and object relatives. In the mismatch subset, we observed a main effect of type and a main effect of reading, as reported in Table 4: subject relatives were comprehended more accurately than object relatives and children with better reading skills were more accurate overall.

**TABLE 4:** Mismatch subset output.

**Fixed effects**	**Estimate**	**SE**	***Z*-value**	***p*-value**
Intercept	1.055	0.208	5.051	< 0.001
Type 2-1	0.504	0.251	2.008	0.044
Reading	0.014	0.006	2.36	0.018
Type 2-1: Reading	–0.003	0.005	–0.61	0.542

*Gray shade indicates significant result.*

**FIGURE 3:**
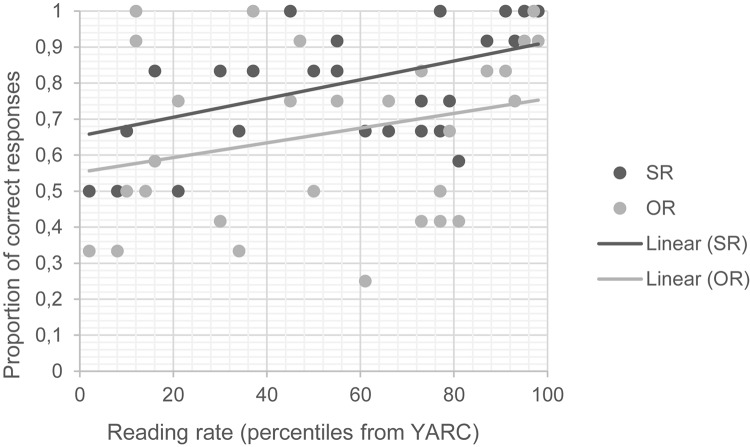
Scatterplot reading – type in the mismatch subset. SR, subject relative; OR, object relatives; YARC, York assessment of reading and comprehension.

This is visualized in Figure 3; the subject relative line is consistently above the object relative line, and the slopes of the lines are both positive and similarly steep.

**FIGURE 4:**
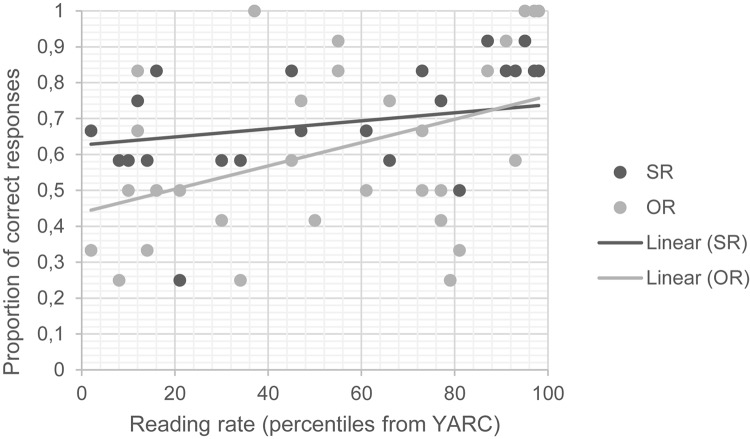
Scatterplot reading – type in the match subset. SR, subject relative; OR, object relatives; YARC, York assessment of reading and comprehension.

In the match subset, instead, we found an interaction between reading and type and a main effect of reading (Table 5). The signs of the estimates can be used to understand the directions of the fixed effects. These show that children with better reading skills were better overall at comprehending relative clauses than poor readers and that the asymmetry between subject and object relative clauses is at its peak in children with lower reading performance, but it gradually decreases in children with better reading skills. The two findings are represented graphically in Figure 4. Both lines have an upward slope, indicating that higher overall reading skills correspond to higher accuracy in relative clauses; the two lines are rather distant in correspondence of lower reading skills, but they get closer and closer with better reading skills, indicating that the asymmetry between subject and object relatives is at its peak for children with poor reading skills, and gradually disappears in skilled readers.

**TABLE 5:** Match subset output.

**Fixed effects**	**Estimate**	**SE**	***Z*-value**	***p*-value**
Intercept	0.817	0.24	3.399	< 0.001
Type 2-1	0.325	0.326	0.996	0.319
Reading	0.013	0.006	2.001	0.045
Type 2-1: Reading	–0.011	0.006	–1.909	0.056

*Gray shade indicates significant result.*

## Discussion

The current study investigated the comprehension of relative clauses in a group of 30 English children (7–11 years) with varying levels of reading skills and it attempts to use this range of measures as a predictor for the accurate comprehension of relative clauses. Reading was included as an independent variable in the model together with *type* of sentence (subject vs. object relative) and *match*, which refers to whether the noun phrases (NP) involved in the action did or did not match in number. Our results can be summarized as follows:

(1) Overall, subject relatives were comprehended better than object relatives.
(2) Sentences with mismatch in number were comprehended more accurately overall than sentences with match in number.
(3) Children with stronger reading skills were more accurate overall than children with lower reading skills
(4) In the sentences in which the noun phrases match in number, the asymmetry between subject and object relatives is at its peak for children with low reading skills, and it gets gradually smaller with the increase of reading skills.

These findings are supported by the existing literature in several respects.

Cross-linguistic evidence shows that children are more accurate in the comprehension of subject relative clauses in comparison to object relative clauses in a large number of studies. In English, this finding replicates seminal work by Brown (1972) and Keenan and Comrie (1979). The finding was also reported in several studies across other languages (Betancort et al., 2009; Friedmann et al., 2009; Theodorou and Grohmann, 2012; Contemori and Belletti, 2014; Hu et al., 2016). As proposed in the introduction, this finding can be explained with reference to the concept of Relativised Minimality (Rizzi, 1990). In subject relatives, the structural configuration which determines intervention of one constituent does not apply, and so the parsing of these sentences is not problematic. In object relatives, instead, the subject of the subordinate clause intervenes in the local relationship occurring between the subject of the main clause and its trace. Intervention poses an additional challenge for the parser and, consequently, object relatives are comprehended less accurately overall than subject relatives.

Second, sentences in which the two verbal arguments mismatch in number (e.g., one being singular and the other being plural) are comprehended more accurately overall than sentences in which these noun phrases match in number (e.g., both are singular or plural). This finding replicates those reported by Adani et al. (2014), who used the same methodology but with different groups of children. In the original study, Adani et al. (2014) reported a facilitation for relative clauses displaying number mismatching in both TD children and children with language impairment. Moreover, the number facilitation has also been reported for other intervention-triggering complex sentences of languages other than English, such as in Italian relative clauses (Volpato, 2012), Dutch wh-questions (Metz et al., 2012) and German object-initial declarative sentences (Adani et al., 2017). In order to explain this effect, we follow Adani et al. (2014), who argued that the higher accuracy observed in mismatch sentences may be the reflection of a facilitatory effect arising from feature dissimilarities on the verbal argument noun phrases (and their agreeing verbs). Specifically, when the intervening subject noun phrase does not share the same number-marking of the moved (object) constituent, intervention effects decrease and, as a consequence, sentence accuracy increases. Number mismatch on the noun phrases entails that the main and embedded verbs are differently inflected depending on which noun they agree with. One possibility is that verbal agreement may be enhancing correct thematic role assignment and thus the interpretation of the sentence. The results presented in this paper reveal that English-speaking primary school children can access detailed representations of number features and that these features can support the parsing of complex structures.

Third, children with stronger reading skills are more accurate in the comprehension of relative clauses. This finding is consistent with previous studies, such as Bar-Shalom et al. (1993), Casalis et al. (2013), and Arosio et al. (2017). In all the previous studies, it was found that children with reading difficulties have additional problems in parsing object relative clauses in comparison to TD children. However, our study crucially differs from these in that, in our sample, children with a reading impairment are not compared to TD children as separate groups. On the contrary, in the present study all children belong to one group and reading skills were modeled as a continuous predictor, thus allowing a more faithful representation of the actual distribution of reading skills in the population (Pennington, 1995).

The fourth effect is the most complex, since it describes the relation among reading skills, number marking, and sentence type. In mismatch sentences, the two main effects of sentence type and reading skills reveal that children with stronger reading skills are more accurate overall than those with poorer reading skills and that subject relatives are comprehended more accurately than object relatives. These effects are independent and do not interact as also demonstrated by the virtually parallel lines reported in Figure 3. On the contrary, in match conditions, it is shown that while stronger reading skills go together with a higher accuracy in the task, reading skills also modulate the levels of accuracy in subject and object relative clauses differently. While the accuracy of poor readers (i.e., those participants who demonstrated a lower performance on the YARC test) is significantly lower for object relative clauses than for subject relative clauses, the accuracy level for both sentence types tends to converge as the reading skills increase. In Figure 4, we reported that in very skilled readers (i.e., the ones who demonstrated highest scores in the YARC test), the asymmetry between subject and object relative clauses is virtually absent. This finding indicates that skilled readers are more accurate than poor readers in parsing non-canonical word order structures such as object relative clauses. It is important to note, however, that the difficulty of object relative clauses is reduced to the extent that their accuracy equals that of subject relatives (for very skilled readers) only in match conditions. This means that this effect emerges only when number-marking is not an overt hint to sentence interpretation. In number mismatching conditions, object relative clauses were persistently more poorly understood than subject relative clauses and the participants’ reading skill level did not modulate their accuracy.

We argue that this result is consistent with a feature-based processing explanation such as the one proposed by Garraffa and Grillo (2008), Grillo (2009), and further elaborated by Adani et al. (2014), which uses Relativised Minimality Rizzi (1990), as a metric to define syntactic complexity. Building on these proposals, in sentences in which number-marking on the verbal arguments was matched (i.e., subject and object noun phrases were either both singular or both plural), the intervention effect is expected to be at its peak. As a consequence, the asymmetry between subject and object relatives is expected to be larger. The asymmetry does indeed appear larger in absolute terms when we look at poor readers (in comparison to the same poor readers with mismatching sentences), suggesting that a formal explanation of the phenomenon holds, at least for the poor readers. The gradual reduction of the asymmetry between subject and object relative clauses as reading skills improve suggests that good to very skilled readers may, arguably, be able to provide a finer-grained representation of the featural make-up of natural language constituents. Their ability to access very detailed linguistic representations may, in turn, reduce the intervention effect observed in object relative clauses, but only in those conditions in which other linguistic markers are not available to support the parsing of the sentence. In the study presented in this paper, this is the context of match conditions, in which disambiguating number-marking was matched (i.e., both verbal arguments were either singular or plural) and, as such, number-marking could not be used as an overt hint to sentence interpretation. Since reading skills correlate with cognitive skills, such as working memory, the asymmetry between subject and object relatives is expected to be smaller in good readers (in line with studies assessing the relation between memory and relative clauses, such as Arosio et al., 2011). The enhanced cognitive skills reported in good readers can in fact allow the processing of features necessary to parse object relatives, thus reducing their difficulty with respect to subject relatives. This is indeed the pattern that we report, with the asymmetry disappearing in very skilled readers. In contrast, the conditions in which number-marking of verbal arguments differs (i.e., mismatching conditions) were understood more accurately than matching ones but the relative difference in accuracy between subject and object relative clauses remains virtually constant across the whole spectrum of reading skills. A plausible explanation of this effect could be that, in mismatch conditions, the role of reading skills is not as central as in match conditions, and possibly because the number feature is already supporting the correct interpretation of complex sentences.

A potential alternative (or complementary) interpretation of these results has to do with exposure. Generally speaking, children with better reading skills tend to read more (Allen et al., 1992). Children that read more are likely to be more exposed to non-canonical word orders, hence they may be less sensitive to the asymmetry between subject and object relative clauses (MacDonald, 2013). This perspective could help us to explain why the input that children receive through text could help to reduce the asymmetry between subject and object relatives in skilled readers, but to do so the account needs to explain why this effect only occurs in those conditions in which number-marking of the verbal arguments corresponds. One possibility is the one advocated in Tsimpli (2014), where it is suggested that exposure plays a predictive role in language acquisition, but only in the acquisition of the so-called “late structures,” i.e., structures that rely heavily on language external resources to be mastered and that are generally more complex. With this approach, it is expected that exposure will play a crucial role in match sentences but not in mismatch sentences, since in match sentences intervention is at its peak and parsing is more complex.

This pattern of results generates some new questions. Are children that are naturally more open to non-canonical word orders developing better reading skills as a consequence of that? Or is it the case that children that tend to read more become more open to non-canonical word orders? The answer may be bidirectional. It may be the case that children that are in general comfortable with complex syntactic structures are those that enjoy reading more, and as a consequence of more time spent reading they become even more open to complex structures. Something similar happens for phonological awareness, where children with good phonological skills tend to acquire reading more quickly, and then the improved attitude to reading allows them to further improve their phonological awareness (Dehaene, 2009). From an applied point of view, it is crucial to stress that even if the phenomenon is indeed bidirectional, one particular direction has important consequences: reading practice is likely to be enhancing the spoken comprehension skills of children. Non-canonical word orders are significantly more frequent in written than in spoken language (Purcell-Gates, 2001; MacDonald, 2013), and extended experience of reading in high skilled readers may be making these children more open to these kinds of complex structures. Previous evidence on different linguistic domains (vocabulary and declarative knowledge) also seem to point in this direction (Cunningham and Stanovich, 1998). This possible interpretation of our result stresses the importance of reading in the development of spoken language.

## Conclusion

This study shows that the asymmetry between subject and object relatives is modulated by reading skills in primary school English children. More specifically, when focusing on sentences with NPs that match in number, the data presented in this paper reveals that object relative clauses are significantly less accurate than subject relative clauses in poor readers but the accuracy gap between the two sentence types gradually decreases as a function of reading skills. While the asymmetry appears at its peak in children that approach the diagnostic criteria for dyslexia, in very skilled readers the asymmetry virtually disappears. Considering that non-canonical word orders are more frequent in written than in spoken language (Purcell-Gates, 2001) and considering that good readers tend to have strong working memory skills (Robertson and Joanisse, 2010), the findings of this study may be interpreted in more than one way. One interpretation could be that children with strong reading (and working memory) skills may provide a finer-grained representation of the featural make-up of nominal phrases and this can help reducing the intervention effect observed in object relative clauses, in which other overt linguistic markers such as number cannot be a hint to correct sentence interpretation (see Garraffa and Grillo, 2008; Grillo, 2009; Adani et al., 2014). A more experience-oriented interpretation of these findings could be that children that enjoy reading and are good readers familiarize more quickly with sentences with non-canonical word order (MacDonald, 2013). This exposure driven effect may be present only in match sentences because only these belong to the so-called “late structures,” that is structures that rely more heavily on language external resources (Tsimpli, 2014).

Further research may help disentangle the relation between these two interpretations. One possibility, for example, would be to control for reading input in parallel experimental trials, as Cunningham and Stanovich (1998) did for the study of the development of vocabulary. This paper may offer a foundation for studies of that kind to be conducted in the near future.

## Ethics Statement

This study was carried out in accordance with the recommendations of “University of Cambridge Ethics Committee” with written informed consent from all subjects. All subjects gave written informed consent in accordance with the Declaration of Helsinki. The protocol was approved by the “University of Cambridge Ethics Committee.”

## Author Contributions

LC and IT designed the study, collected the data, and prepared the first draft. FA created the main experimental task and revised the manuscript. All authors approved the final version of the manuscript and agreed to be accountable for all aspects of the work in ensuring that questions related to the accuracy or integrity of any part of the work are appropriately investigated and resolved.

## Conflict of Interest Statement

The authors declare that the research was conducted in the absence of any commercial or financial relationships that could be construed as a potential conflict of interest.

## Footnotes

1 For what concerns the CNRep norms are available up to the age of 9;11. For the six children in our sample above this age, percentiles were calculated looking at the closest band and approximating by defect. For example, UK7, with a raw score of 31, was between the 10th and the 25th percentile for an age of 9;11. Being the child 10;04, the score of 31 was then coded as 10th percentile.

## Appendix

# Selection of the random structure.

M1 < - glmer[correct ∼ match^*^type^*^yarcRATE.cent + (1|part) + (1|item)].

M2 < - glmer[correct ∼ match^*^type^*^yarcRATE.cent + (match|part) + (1|item)].

M3 < - glmer[correct ∼ match^*^type^*^yarcRATE.cent + (type|part) + (1|item)].

M4 < - glmer[correct ∼ match^*^type^*^yarcRATE.cent + (match + type|part) + (1|item)].

M5 < -glmer[correct ∼ match^*^type^*^yarcRATE.cent + (match^*^type|part) + (1|item)].

M6 < -glmer[correct ∼ match^*^type^*^yarcRATE.cent + (1|part) + (match|item)].

M7 < -glmer[correct ∼ match^*^type^*^yarcRATE.cent + (1|part) + (type|item)].

M8 < -glmer[correct ∼ match^*^type^*^yarcRATE.cent + (1|part) + (match + type|item)].

M9 < -glme[correct ∼ match^*^type^*^yarcRATE.cent + (1|part) + (match^*^type|item)].

M10 < -glmer[correct ∼ match^*^type^*^yarcRATE.cent + (match|part) + (match|item)].

M11 < -glmer[correct ∼ match^*^type^*^yarcRATE.cent + (match|part) + (type|item)].

M12 < -glmer[correct ∼ match^*^type^*^yarcRATE.cent + (match|part) + (match + type|item)].

M13 < -glmer[correct ∼ match^*^type^*^yarcRATE.cent + (match|part) + (match^*^type|item)].

M14 < -glmer[correct ∼ match^*^type^*^yarcRATE.cent + (type|part) + (match|item)].

M15 < -glmer[correct ∼ match^*^type^*^yarcRATE.cent + (type|part) + (type|item)].

M16 < -glmer[correct ∼ match^*^type^*^yarcRATE.cent + (type|part) + (match + type|item).]

M17 < -glmer[correct ∼ match^*^type^*^yarcRATE.cent + (type|part) + (match^*^type|item)].

M18 < -glmer[correct ∼ match^*^type^*^yarcRATE.cent + (match + type|part) + (match + type|item)].

All models contain: data = ukstudy2, family = binomial, control = glmerControl(optimizer = “bobyqa”, nAGQ = 10), na.action = na.omit

Only M1, M2 and M3 converged. M3 has the smallest AIC.

# Df AIC BIC logLik deviance Chisq Chi Df Pr( > Chisq).

# M1 10 1589.0 1641.7 -784.48 1569.0.

# M2 12 1592.9 1656.2 -784.47 1568.9 0.0172 2 0.9915.

# M3 12 1587.4 1650.7 -781.70 1563.4 5.5279 0 < 2e-16^∗∗∗^.
